# Myelin Oligodendrocyte Glycoprotein-Antibody Associated Disease: An Updated Review of the Clinical Spectrum, Pathogenetic Mechanisms and Therapeutic Management

**DOI:** 10.3390/antib13020043

**Published:** 2024-05-17

**Authors:** Panagiotis Gklinos, Ruth Dobson

**Affiliations:** 1 First Neurology Department, Eginition University Hospital, Medical School, National and Kapodistrian University of Athens, 11528 Athens, Greece; 2 Centre for Preventive Neurology, Wolfson Institute of Population Health, Queen Mary University of London, London EC1M 6BQ, UK; ruth.dobson@qmul.ac.uk

**Keywords:** myelin oligodendrocyte glycoprotein, antibody, autoimmune diseases, neuroinflammation, neuromyelitis optica spectrum disorders

## Abstract

Clinical syndromes associated with antibodies against myelin oligodendrocyte glycoprotein (MOG) are now recognized as a distinct neurological disease entity, and are gaining increasing attention. The pathogenic mechanisms underlying MOG-antibody disease (MOGAD) remain incompletely understood. Case series, facilitated by registries, and observational studies over the past few years have shed increasing light on the clinical aspects and therapeutic approaches of MOGAD. MOGAD may manifest with a variety of clinical syndromes, including acute disseminated encephalomyelitis (ADEM), autoimmune encephalitis, optic neuritis (ON) and transverse myelitis (TM). MOGAD can be either monophasic or relapsing. This review aims to provide a comprehensive updated description of the clinical spectrum, paraclinical features, and prognosis of MOG-antibody disease, as well as summarize its therapeutic considerations. Randomized clinical trials, standardized diagnostic criteria and treatment guidelines are the steps forward.

## 1. Introduction

Myelin oligodendrocyte glycoprotein (MOG) is a protein expressed exclusively on the surface of oligodendrocytes in the central nervous system (CNS) [1,2,3]. Although its precise biological role remains unclear, it is believed that it contributes to the completion, maintenance, and structural integrity of myelin, as well as playing a role in cell-to-cell communication [3]. Until recently, it was speculated that MOG could be a potential autoantigen in multiple sclerosis (MS); however, this is not the case, as neuroinflammatory disease associated with MOG antibodies has a different clinical phenotype from MS with a range of manifestations [4].

Over recent decades, the development of highly sensitive and specific cell-based assays for MOG antibody detection has allowed us to identify a subset of patients with a clinical phenotype distinct from MS and neuromyelitis optica spectrum disorders associated with aquaporin-4 antibodies (AQP4 + NMSOD) [5,6]. With this, MOG-antibody associated disease (MOGAD) has evolved into a discrete autoimmune, neuroinflammatory disease entity with a broad clinical spectrum which continues to evolve as our understanding increases. Clinical phenotypes associated with MOGAD may overlap with those observed in MS and AQP4 + NMSOD, highlighting the importance of recognizing key diagnostic approaches, enabling neurologists to make the correct diagnosis and employ the most appropriate treatment strategies.

In this review, we provide an updated overview of the clinical, radiological and biochemical characteristics of MOG-antibody associated disease, discuss the prognosis, highlight the differences from MS and AQP4-NMOSD, and explore the acute and preventive therapeutic options. 

## 2. Pathogenesis

MOG is a minor constituent of myelin, and is found on the surface of oligodendrocytes [7,8,9,10,11,12]. It has a length of 245 amino acids (AA) and a molecular weight of 26–28 kDa. MOG has a β-strand structure that spans the cell membrane twice, and has an IgG-like domain on the extracellular N-terminal end [11,12]. Belonging to the immunoglobulin superfamily, MOG is highly immunogenic. While its exact role remains incompletely understood, it likely serves as a cell adhesion molecule, helps regulate microtubule stability, and modulates interactions within the myelin immune system [11,12]. Positioned on the outermost layer of the myelin sheath in the central nervous system (CNS), MOG becomes a potential target for MOG antibodies. These antibodies trigger demyelination in experimental autoimmune encephalomyelitis animal models immunized with MOG [11]. However, it is important to note that most human MOG antibodies do not recognize rodent MOG, limiting the applicability of many rodent studies to human MOGAD [11,12]. Interestingly, human MOG antibodies that do recognize rodent MOG, when injected intrathecally in rodents alongside myelin-reactive T cells, were observed to be directly pathogenic [12].

**Figure 1:**
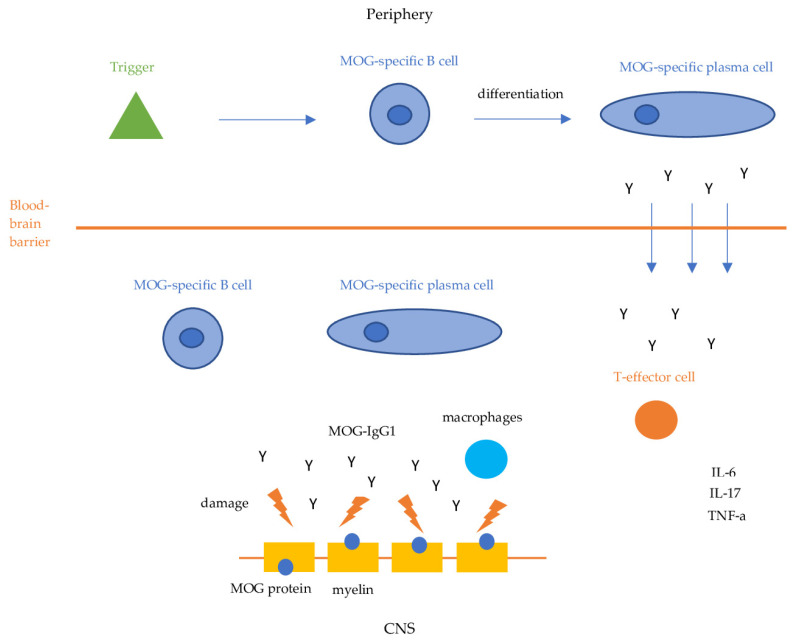
MOGAD pathogenesis. IL-6: Interleukin-6, IL-17: Interleukin 17, TNF-a: Tumor Necrosis Factor-a.

Our current understanding of the pathophysiology of MOGAD is summarized in Figure 1. Although the pathogenesis of MOGAD is increasingly being studied, the precise mechanisms remain unclear. It has been proposed that the initial trigger might be an infection, which could induce autoimmunity through various mechanisms such as molecular mimicry, bystander activation or polyclonal activation of B-cells. However, no specific pathogen has been identified so far, and many people presenting with MOGAD do not report antecedent symptoms suggestive of infection. MOG-specific B cells, plasma cells and their products (MOG Abs) activate MOG-specific effector T cells via CNS resident antigen-presenting cells (APC) [7,8]. Subsequently, B cells, plasma cells and autoantibodies directed against the MOG antigen cross the blood–brain barrier (BBB) and react with their antigens inducing their pathogenic effects [9,10,11,12]. Anti-MOG antibodies (IgG1) bind MOG expressed on the surface of myelin and oligodendrocytes, damaging myelin sheaths, and leading to demyelination through antibody-dependent cellular cytotoxicity or complement activation [9,10].

Simultaneously, MOG-specific B cells and plasma cells activate MOG-specific CD4 T cells and macrophages which mediate an inflammatory response by activating proinflammatory cytokines such as IL-6, IL-17, and TNF-a, resulting in further inflammation and demyelination [11,12]. Perivenous and confluent white matter demyelination, MOG-dominant myelin loss, intracortical demyelination, predominant CD4+ T-cell and granulocytic inflammation, complement deposition within active white matter lesions, partial axonal preservation, and reactive gliosis are the pathological hallmarks of MOGAD [11,12]. The central role of MOG-specific CD4 T cells in the pathogenesis of MOGAD has been demonstrated by studies that reveal their presence in inflammatory lesions of MOGAD patients [11,12].

## 3. Clinical Spectrum

### 3.1. Frequency and Demographics

Limited epidemiological data on MOGAD exist, primarily due to the recent discovery of myelin oligodendrocyte glycoprotein immunoglobulin G (MOG-IgG) in 2007, with widespread testing becoming available only years to a decade later. As a result, initial reports on the epidemiology of MOGAD may have underestimated its frequency. The incidence and prevalence remain largely unknown, though European studies indicate an incidence ranging from 1.6 to 3.4 per 1,000,000 person-years [13,14]. The first attack of MOGAD can occur in any decade of life. Whilst mean age of onset is around the beginning of the fourth decade of life [6,13,14], MOG antibodies are not uncommonly associated with first demyelinating events in children, particularly acute demyelinating encephalomyelitis (ADEM) and optic neuritis (ON). In contrast to AQP4-NMOSD, which is more common in African American and Afro-Caribbean individuals, no particular racial preponderance has yet been identified in MOGAD [15,16,17]. Among young children (<10 years old), there is no difference between males and females; however, there is a slight female predominance in older children and adults (1.5:1), which is significantly less than the observed female predominance in AQP4 + NMOSD [16,18].

MOGAD can occur at any age; however, the incidence is higher in children compared to adults when compared to MS and AQP4 + NMOSD [19], with MOGAD being a more frequent cause of a first demyelinating episode in children than in adults. More specifically, MOG antibodies were detected in 18–39% of children with a first demyelinating episode, including ADEM and ON across different studies, compared to only 1.2–6.5% in adults [20,21,22,23,24,25,26,27]. Several studies have consistently found that pediatric MOGAD patients, particularly those under the age of five, most commonly present with ADEM, while children older than 11 tend to present more often with ON or TM [28,29,30]. Approximately 40% of children with ADEM have been found to be seropositive for MOG antibodies [2]. Children typically face a lower risk of experiencing a relapsing course compared to adults, prompting questions in clinical practice about the necessity of long-term therapeutic intervention and disease monitoring [31]. The high prevalence of MOG antibodies in children with demyelinating events potentially indicates the age-dependent mechanisms of demyelination.

### 3.2. Clinical Syndromes

MOGAD associated syndromes are summarized in Table 1. Similarly, to frequency, the disease clinical phenotype largely depends on the age of the patient. Children are more prone to develop an encephalopathic syndrome (50%), which manifests mostly as ADEM with or without optic neuritis. On the other hand, adults most frequently present with optic neuritis (50%) or transverse myelitis (30%) [5,21,23,25,32].

#### 3.2.1. Optic Neuritis

Optic neuritis is the most common initial manifestation of MOGAD in adults (50%). Approximately 80% of MOGAD patients will develop optic neuritis at some point over the course of the disease. It is characterized by varying degrees of vision loss, and is nearly always accompanied by eye pain that worsens with eye movement, which often precedes vision loss. As in AQP4 + NMOSD, vision loss in MOGAD is more severe compared to MS, and is typically central. Caution is warranted in pediatric patients where eye pain can be mistaken for headache and vision loss can be significantly underreported [33].

ON is often bilateral (30–50%), and is associated with optic disc edema (86%). These features allow differentiation from MS, where simultaneous bilateral disease is extremely rare [34]. Optic disc edema is more severe than in MS and AQP4 + NMOSD, and can even result in peripapillary hemorrhages. The optic nerve lesion is typically long and affects the anterior optic pathway.

Although severe, ON in MOGAD patients responds well to steroid treatment, and recovery is usually good, with only 6–14% experiencing a residual visual acuity of 20/200 or worse [35,36]. However, residual optic disc pallor and retinal nerve fiber layer (RNFL) thinning on optical coherence tomography (OCT) is common in ON associated with MOGAD, making subsequent attacks likely to be particularly severe [37,38].

#### 3.2.2. Transverse Myelitis

Around 30% of adults with MOGAD will present with transverse myelitis. Symptoms include weakness that can result in paraparesis or quadriparesis, and sensory loss below the level of the lesion with a sensory level across the trunk and prominent bladder (urinary retention requiring catheterization) and bowel involvement [39,40,41]. Males also present with erectile dysfunction. Sphincter dysfunction tends to occur more frequently in MOGAD myelitis when compared to MS and AQP4 + NMOSD [40]. This heightened prevalence is likely attributed to the increased occurrence of lesions affecting the conus medullaris in MOGAD. Residual bowel, bladder, and erectile dysfunction are frequently observed and can often be more pronounced than any remaining motor deficits [41]. MOGAD myelitis can be very severe at nadir, similarly to AQP4 + NMOSD myelitis (Expanded Disability Status Scale (EDSS) ≥ 7 in over 30%) [41]. In roughly three-quarters of cases, the lesions detected during myelitis on sagittal T2-weighted magnetic resonance imaging (MRI) extend over three or more vertebral segments, a condition termed longitudinally extensive transverse myelitis (LETM). Typically, these lesions are centrally located within the spinal cord, in contrast to MS, where the lesions are mostly located within the posterior aspect of the spinal cord and are almost always less than three vertebral segments. In addition, lesions in MOGAD myelitis are frequently confined to the grey matter, resulting in the characteristic H-shape, as opposed to AQP4 + NMOSD, where lesions are centrally located, but involve both the grey and the white matter. Although LETM is very common in MOGAD, some patients may present with both a LETM and a short spinal cord lesion, while a minority may only have a short lesion [39,40]. Transverse myelitis, particularly when affecting the motor tracts, has been shown to be the most significant predictor of disability [32]. Although, one of the most severe manifestation of MOGAD, transverse myelitis responds better to steroid treatment than in MS or in NMOSD, with only 6% requiring walking aid at last follow up [39]. In a study comparing outcomes of myelitis attacks between AQP4 + NMOSD and MOGAD, the median EDSS at myelitis recovery was 3.0 (range 1.0–8.0) for AQP4 + NMOSD and 1.8 (range 1–8.0) for MOGAD. Notably, only 7% of patients in the MOGAD group had an EDSS ≥ 6 at recovery, compared to 44% in the AQP4 + NMOSD group [40].

#### 3.2.3. ADEM

ADEM is defined as a clinical syndrome marked by an initial polyfocal episode within the CNS, presumed to result from demyelination [42]. ADEM typically features encephalopathy, which is not attributable to fever, systemic illness, or postictal phenomena. It typically manifests with MRI abnormalities characterized by large, poorly demarcated lesions within the white matter, sometimes accompanied by lesions in the gray matter [42]. ADEM is the most common initial manifestation of MOGAD in children under 11 years old (more than 50%). It presents as an acute or subacute, widespread inflammation of the CNS, involving the brain and sometimes the spinal cord as well. Symptoms include drowsiness, confusion, disorientation, and impaired speech, as well as focal neurologic signs. A study by Wendel et al. showed that 59% of children with MOGAD who initially presented with ADEM had a monophasic disease course [43]. The same study showed that MOG-IgG titers were associated with the risk of a relapse, with a statistically significant decrease in MOG-IgG titers during the first and second years in those with monophasic disease compared to those who relapsed. A significantly higher percentage of seroconversion to MOG-IgG-negative was observed in monophasic patients (62% in the second year, compared to 0% in the relapsing group). In contrast, in adults, MOGAD encephalomyelitis is rare, accounting for only 5% of the cases.

#### 3.2.4. Fluid Attenuated Inversion Recovery (FLAIR)-Hyperintense Lesions in Anti-MOG-Associated Encephalitis with Seizures (FLAMES)

A distinct clinical syndrome called FLAMES has been described in 2017 by Ogawa et al. [44]. More specifically, they reported four patients with unilateral cortical encephalitis and associated seizures who were MOG-antibody positive. Since then, FLAMES has been repeatedly reported in several case reports and case series [45,46,47,48,49,50]. Interestingly, it is reported that a subset of patients with FLAMES had leptomeningeal enhancement, indicating meningeal involvement, which goes beyond the limits of cortical inflammation. Some patients had only leptomeningeal enhancement, and only a few or no cortical hyperintensities, described in a review from Mayo Clinic and referred to as FLAIR-variable Unilateral Enhancement of the Leptomeninges (FUEL) [51]. Patients with FLAMES predominantly present with focal onset seizures, which can then progress to secondary generalized, tonic-clonal seizures. Headaches, fever, and cortical symptoms referable to the corresponding lesion are very common. Headaches may be severe, and can have increased intracranial pressure characteristics. Imaging features of FLAMES include typically unilateral cortical high intensity signal, mostly within the frontal and parietal lobe, though bilateral cortical hyperintensities have also been described [52]. Prior to the description of FLAMES, encephalitis has been historically linked to ADEM in patients with MOGAD. Although rare, some cases of hemiencephalitis with associated seizures have been reported [5,44].

#### 3.2.5. MOGAD and N-Methyl-D-Aspartate-Receptor (NMDAR) Encephalitis Overlap

In recent years, an overlapping syndrome of MOGAD and NMDAR encephalitis has been described. Its frequency and pathogenetic mechanisms remain largely unknown. It is speculated that the overlapping syndrome can be a result of autoimmunity against oligodendrocytes, since they can express NMDAR-receptors as well, a result of secondary immune reaction or a bystander phenomenon [2,53]. Clinically, most patients present with atypical NMDAR encephalitis. For example, a patient who presents with NMDAR encephalitis, but with associated optic neuritis or transverse myelitis, should alert the clinicians and should be tested for MOG-antibodies as well. The concurrence of NMDAR with MOGAD suggests a more aggressive course, and requires more aggressive treatment than MOGAD alone, as well as cancer screening since NMDAR-encephalitis is known to be linked with specific cancers. Vice versa, patients with MOGAD and atypical features such as behavioral or psychiatric symptoms should be screened for NMDAR-encephalitis as well.

#### 3.2.6. Other Demyelinating Syndromes

Although rare, MOGAD can present as brainstem demyelination with diplopia or other brainstem syndromes and/or NMOSD without anti-AQP4 antibodies [54]. Isolated brainstem or cerebellar syndromes are not a common presentation of MOGAD, but rather occur as part of a multifocal CNS attack, along with other common MOGAD syndromes such as ON or TM. Symptoms can vary from diplopia to ataxia, and depend on the infratentorial region that is affected [55]. Low titers of anti-MOG-IgG and isolated brainstem or cerebellar syndrome should prompt the neurologist to consider alternative diagnoses.

Since MOGAD can manifest with ON or LETM, it is reasonable that it accounts for a large proportion of the AQP4 seronegative NMOSD cases. Noticeably, there have been scarce reports of MOGAD manifesting with an area postrema syndrome including nausea, vomiting, and hiccups [56,57,58,59]. This can be either in isolation or as a component of a multifocal CNS attack with lesions within the area postrema at the caudal end of the fourth ventricle.

In cases where MOGAD presents with an atypical syndrome, it is crucial to identify distinguishing features from other similar conditions such as MS or AQP4 + NMOSD [60]. Lack of dissemination in time at onset either clinically or from imaging (black holes in MS) is a key feature. In terms of imaging, MOGAD-associated ON is often bilateral, and involves the anterior optic pathway, while AQP4 + NMOSD-associated ON involves the posterior optic pathway, including the optic chiasm. ON in MS is rarely bilateral. LETM is another important clinical feature that helps differentiate NMO from MS. While MS-associated myelitis rarely extends beyond three vertebral segments, and usually affects the posterior part of the spinal cord, MOGAD-associated myelitis typically extends over three vertebral segments, forming a characteristic “H” shape due to its confinement to the grey matter. This is typical for MOGAD LETM, and differs from AQP4 + LETM, where the lesion is again central but affects both the grey and the white matter. Furthermore, brain lesions in MOGAD are typically fluffy, poorly demarcated, and located in juxtacortical areas, resembling lesions seen in ADEM, as opposed to MS where lesions are periventricular and sharply demarcated.

## 4. Imaging Characteristics and Biochemical Biomarkers

### 4.1. Imaging Studies

The brain MRI, excluding the optic nerve, is abnormal in approximately 45% of the cases [5]. Parenchymal lesions are fluffy, poorly demarcated, and similar to those observed in ADEM. Both the white matter and the deep grey matter, including unilateral or bilateral thalamic or basal ganglia T2 hyperintensities, can be involved. Infratentorial lesions are also common, with extensive involvement of the middle cerebellar peduncles and the pons [61,62]. Lesions within the middle cerebellar peduncles in MOGAD are larger and less well demarcated compared to MS, where lesions are smaller and well demarcated. Cortical hyperintensities may also be found. Dawson fingers and ovoid lesions adjacent to the lateral ventricles are less frequently observed [63]. It is important to know, however, that brain MRI can be entirely normal, particularly in patients presenting with LETM.

The spinal cord is involved in approximately half of the cases, and some patients have more than one spinal cord lesion. Characteristic findings include LETM spanning in more than three vertebral segments, as well as lesions confined to the grey matter (H-shaped lesions) [64]. However, more than half of the patients with spinal cord lesions have short lesions, less than three vertebral segments. Conus medullaris involvement is considered very common, and contrast enhancement is observed in nearly half of the cases [64]. Finally, it has been described that children with severe radiological abnormalities can have a relatively mild clinical presentation that does not correspond to the MRI findings, similarly to other causes of ADEM [65].

An optic nerve MRI can show unilateral or bilateral high T2 signal, which is frequently extensive, involves the anterior optic pathways extending up to the fundus (which probably explains the frequent optic disc edema), and is associated with optic nerve swelling [17]. Contrast enhancement is seen in almost all cases of ON, and extends in >50% of the length of the optic nerve. In some cases, enhancement is confined to the optic nerve sheath (optic perineuritis) [17].

### 4.2. Biomarkers

Recommendations on MOG antibody testing are summarized in Table 2.

Cell-based assays should be used to measure MOG-antibody titers in the serum. In assay comparison studies, the live cell-based assay demonstrated slightly superior performance compared to inactivated cell-based assays [66,67]. Protein-based enzyme-linked immunosorbent assay (ELISA) MOG-IgG tests lack clinical utility, and often yield unreliable results. When evaluated through a cell-based assay utilizing MOG in its full-length conformational form, MOG-IgG serves as a highly specific biomarker for MOGAD, with specificity ranging from 97.8 to 100 percent [66]. However, the positive predictive value (true positives divided by total positives) varies more widely, ranging from 72 to 94%. This variability in positive predictive value is influenced by test-ordering practices and disease prevalence. Testing for this rare disease in settings with a low probability of occurrence increases the likelihood of false positivity. MOG-antibody testing should be done only in cases with clinical or imaging features in keeping with MOGAD, such as bilateral ON, LETM or in atypical CNS demyelinating syndromes, to avoid false positive results and overdiagnosing. Uniformly testing MOG-IgG for patients with typical clinical features and MRI findings of MS is not recommended. The presence of a notable background rate of low-titer MOG-IgG (e.g., 1:20 to 1:40) positivity in the general population underscores the challenge of false positive titers in the assessment for MOGAD. In a study analyzing 1260 clinical samples, out of 92 positive MOG-IgG results, it was found that about half of the low titers (1:20 to 1:40) were false positives [68]. Meanwhile, at moderate titers (1:100), 18% were false positives, and at higher titers (≥1:1000), there were no false positives detected. In another study involving 2107 consecutive adult inpatients assessed for various neurological conditions at a German hospital, MOG antibody positivity was detected in 1.2 percent of cases, typically at low titers [69]. However, only 0.2 percent were confirmed to have true MOG antibody-associated disease (MOGAD). Notably, many patients with initially low positive titers subsequently tested negative in follow-up assays. A study of MOG-IgG frequency in a large MS cohort showed that only 0.3% of MS patients were also positive for MOG-IgG [26]. These findings are consistent with another study focusing on progressive multiple sclerosis (MS) phenotypes [70]. Among 290 samples collected from MS patients, only one patient with low titers of MOG-IgG was identified. This suggests a rare occurrence of MOG antibody positivity in patients with progressive MS phenotypes.

There is no consensus on CSF MOG-antibody testing, although it looks that most of the CSF positive cases are also seropositive. There are, however, few cases in which CSF is positive and serum negative; hence, CSF testing can be used in highly suspected cases with negative serum [10,71]. Around half of the patients have CSF pleocytosis (lymphocytes and monocytes) and elevated CSF protein. White cells count is generally higher than in MS [18]. Oligoclonal bands (OCBs) are unusual, and are found in less than 15% of cases [18]. Where OCBs are present, careful clinical and neuroradiological review should be undertaken in order to provide diagnostic certainty.

Table 3 provides an overview of the main demographic, clinical, and paraclinical characteristics of MOGAD compared to NMOSD and MS.

## 5. Diagnostic Criteria

Recently, the first set of diagnostic criteria for MOGAD was proposed by an international panel of experts based on a thorough literature review and a structured consensus process [72]. These require the presence of a core clinical demyelinating event, including ON, TM, ADEM, cerebral monofocal or polyfocal deficits, brainstem or cerebellar deficits or cerebral cortical encephalitis, often with seizures, along with a clearly positive MOG-IgG test and exclusion of a better diagnosis, including MS. In cases of a low positive result, positivity without a reported titer, or negative serum testing with cerebrospinal fluid (CSF) positivity, additional supportive MRI or clinical evidence is essential for diagnosis. For optic neuritis, supportive features include bilateral simultaneous clinical involvement, longitudinal optic nerve involvement (>50 percent length of the optic nerve), perineural optic nerve sheath enhancement, and optic disc edema. In cases of myelitis, supportive characteristics include LETM, central cord lesion or axial H-sign on imaging, and conus lesion. For brain, brainstem, or cerebral syndrome, multiple ill-defined T2-hyperintense lesions in supratentorial and often infratentorial white matter, deep gray matter involvement, ill-defined T2-hyperintensity involving pons, middle cerebellar peduncle, or medulla, and cortical lesion with or without lesional and overlying meningeal enhancement are considered supportive evidence. The panel emphasizes the significance of conducting MOG-IgG testing within a suitable clinical framework to improve the positive predictive value of the test. False positive diagnoses can arise from MOG-IgG seropositivity at lower titers in other demyelinating diseases, such as multiple sclerosis (MS).

## 6. Disease Course and Prognosis

Studies have shown that MOGAD disease course is heterogenous. In the past, it was believed that MOGAD is a monophasic disease. However, we now know that MOGAD can be relapsing in approximately 35% of the cases [30,73].

Relapses typically manifest within the first 6 months after the initial attack, rather than later, and they frequently occur following the tapering or cessation of oral steroid medication [72]. Despite the type of the first attack, relapses tend to manifest as option neuritis in most of the cases [13,14].

Timing of the first relapse has been shown to be important for the risk of future relapses. A retrospective study of 289 adults and children with MOGAD found that relapses within 12 months of onset were linked to a higher likelihood of experiencing further relapses beyond that initial period, whereas a relapse within 90 days appears not to indicate a chronic inflammatory process in young pediatric-onset disease [74].

High MOG-antibody titers on presentation and MOG-antibody persistence favor a relapsing course and worse outcomes, whereas low titers and seroconversion to negativity indicate increased likelihood of a monophasic and milder disease course. Interestingly, it has been shown that seroconversion to negativity 12 months after the first event is associated with a 90% likelihood of experiencing a monophasic course [13,14]. However, MOG-antibody titers can rise and become positive again, even after a few years of being negative; hence, it is suggested testing them on an annual basis even after seronegativity, since seroconversion to positivity can be associated with a relapse [15]. At the moment, there is no consensus on the frequency of MOG-antibody testing; however, 6-monthly and 12-monthly testing are most commonly adopted in clinical practice [72]; this is pragmatic in many cases. MOG antibody monitoring may help clinical decision making around treatment strategies and ongoing immunosuppression; however, further evidence is required to develop more definitive guidelines around this.

## 7. MOGAD and Pregnancy

Since MOGAD can affect women of childbearing age, it is crucial to comprehend the disease’s effects on pregnancy and the postpartum period, as this can greatly influence family planning decisions. Although data is limited, a systematic literature review concluded that disease activity appears to be attenuated during pregnancy in MOGAD patients, with an increased risk of relapse during the postpartum months [75].

An increase in relapse risk during the postpartum period might stem from shifts in immune tolerance during pregnancy that reverse after childbirth. Additionally, it is plausible that discontinuation or insufficient immunosuppression could contribute to postpartum attacks, indicating the need for proactive measures by clinicians post-childbirth to reinstate adequate suppression of the autoimmune response through intensive treatment.

However, additional clinical research is warranted on the management, progression, and outcomes of MOGAD in women of childbearing age.

## 8. Treatment

### 8.1. Acute Treatment

Currently, there are no evidence-based guidelines on the management of the acute attack of MOGAD, since there have been no randomized-controlled clinical trials conducted. However, as in other demyelinating conditions, the soonest the treatment is initiated, the better the outcomes are. It has been shown that MOGAD clinical syndromes are very responsive to steroid treatment [9,43]. Patients with severe symptoms can improve dramatically only after a short course of intravenous steroids. It is recommended that a 3–5-day course of 1g of intravenous methylprednisolone should be given depending on the severity and the response of the attack. In severe cases, or in those who will not improve after 5 days, escalation to plasma exchange (5–7 cycles on alternative days), intravenous immunoglobulins (total of 2 g/kg over 2 or 5 days), or plasma exchange followed by intravenous immunoglobulins can be considered [15]. A steroid taper over 3–6 months should be considered in all cases, as prevention of early relapses has been shown to reduce long-term disability [76]. In most cases, it is anticipated that symptoms will subside, and the patient will improve significantly.

In around 20% of the cases, there might be residual symptoms after the attack, which can cause long term disability. However, it is important to start treatment as soon as possible, as this has been proven to be a crucial factor for the outcome [77]. There is no need to wait for the serum MOG-antibody results, as these do not determine treatment options in the acute setting. Experts have suggested that a slow, gradual taper of oral glucocorticoids over a span of several months might decrease the likelihood of early relapse [76]. A slow taper of glucocorticoids might prove beneficial for patients with relapsing disease who are initiating maintenance attack-prevention immunotherapy, as this treatment often takes weeks to months to exert its full effect. Nonetheless, more research is necessary to fully understand the advantages and drawbacks of this approach, especially for individuals experiencing frequent relapses, given the considerable potential adverse effects associated with prolonged oral glucocorticoid therapy.

### 8.2. Preventive Treatment

Although randomized-controlled clinical trials have not been conducted, patients with MOGAD who are not considered high risk for relapse are given oral steroids (prednisolone 1g/kg/day) for 3 months followed by prolonged tapering (3 months). It has been shown that prolonged tapering is essential to prevent disease rebound activity, which mostly occurs in doses less than 20 mg/day or shortly after steroid cessation [9,76]. Risk factors for a relapse include initial presentation with transverse myelitis or encephalitis, high titer of MOG-antibodies, and incomplete recovery [73].

In more severe cases, it is recommended to continue steroid treatment for either 6 or 9 months, and then slowly wean it off. The exact duration of steroid treatment depends again on the above-mentioned factors, and is largely a clinical decision since there are no standardized protocols. The role of MOG-antibody titer monitoring every 6–12 months is not clear; however, it can impact therapeutic decisions, as seroconversion to negativity has a 90% negative prognostic value for a relapse. Consequently, the cessation of steroids could be considered safe at that stage.

**Figure 2:**
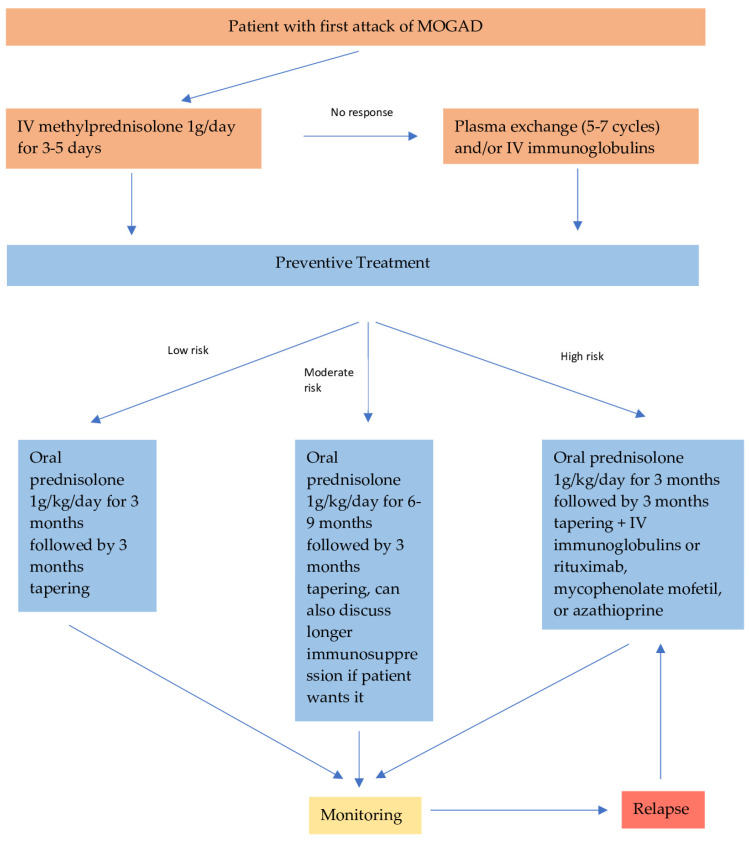
Recommended treatment algorithm for patients with MOGAD.

Patients with very poor prognostic factors on presentation, such as transverse myelitis or encephalitis with severe disability and/or very high MOG-antibody titer should be offered additional immunosuppressive treatment, in addition to oral steroids as preventive treatment. These patients have an increased chance of relapsing; therefore, they require a more aggressive approach. Data from retrospective studies have shown that intravenous immunoglobulin is the most effective preventive treatment option in MOGAD, with around 70% of the patients achieving remission [78,79]. Rituximab is the second most effective option, with 50% relapse-free patients, followed by mycophenolate mofetil (47%) and azathioprine (39%) [9,76,77,78,79,80]. Interestingly, rituximab is not as effective as it is in AQP4 + NMOSD. In Figure 2, we suggest a treatment algorithm depending on the risk factors of a patient with MOGAD.

Finally, new agents are currently being investigated for MOGAD. Tocilizumab, an anti-Interleukin-6 (IL-6) receptor monoclonal antibody, plays a crucial role in B cell maturation and antibody production. Administered at a dose of 8 mg/kg via IV infusion every four weeks, it has shown promise in reducing annualized relapse rates in patients with relapsing MOGAD, as suggested by limited retrospective observational studies [81,82]. Rozanolixizumab is a humanized IgG4 mAb targeting the neonatal Fc receptor (FcRn). FcRn is responsible for IgG recycling intracellularly, and inhibiting it leads to accelerated elimination of IgG. Rozanolixizumab, hence, reduces plasma IgG levels. Phase 3 trials have shown its efficacy and safety in myasthenia gravis, and the drug is now tested for MOGAD as well [83]. Additionally, clinical trials assessing the efficacy of satralizumab, which prevents IL-6 pro-inflammatory signaling pathway and rituximab, an anti-CD20 monoclonal antibody are currently ongoing.

## 9. Conclusions

The field of MOGAD is rapidly evolving. The description of new clinical syndromes associated with the disease has expanded its clinical spectrum, and helped us better recognize and diagnose the disease early. Standardized diagnostic criteria, as well as treatment guidelines, are still lacking, however. In the light of anticipated randomized clinical trials, disease-specific biomarkers as treatment response measures or outcome measures are needed. Although our understanding of the disease has improved significantly, more research on its pathogenesis is required to shed additional light on the possible mechanisms and the causes of a relapsing course, as well as to define new treatment targets.

## Figures and Tables

**Table 1:** Presenting clinical syndromes.

Clinical Syndrome		Features	
Optic Neuritis (ON)	Most common presentation in adults (50%)	Often bilateral Long lesions within the anterior optic pathway	Good recovery, but with excessive optic disc edema and RNFL thinning
Transverse Myelitis	Initial presentation in 30% of adults	Motor, sensory, or autonomic symptoms	Significant predictor of disability, but more steroid responsive than NMOSD and MS
ADEM	Initial presentation in 50% of children. Very rare in adults (5%)	Presenting symptom is seizures in 40% of the children with ADEM and MOGAD	Increased risk of post-ADEM epilepsy
FLAMES	Unilateral cortical hyperintensities in the FLAIR sequence with associated seizures	Focal seizures which can progress to generalized, headache, encephalopathy	Meningeal involvement can be present
MOGAD & NMDAR overlapping syndrome	MOG antibodies and NMDAR-antibodies co-existence	Patients present with atypical NMDAR encephalitis or atypical MOGAD	Requires more aggressive treatment than MOGAD alone and cancer screening
Multifocal CNS attack	Can involve the optic nerve, spinal cord, brainstem and cerebellum	Vision loss, motor/sensory symptoms, ataxia, diplopia	
NMOSD syndrome	ON, LETM	Rarely intractable nausea and vomiting	AQP4 seronegative

ON: optic neuritis; LETM: longitudinal extensive transverse myelitis, ADEM: acute disseminated encephalomyelitis; FLAMES: FLAIR-hyperintense Lesions in Anti-MOG-associated Encephalitis with Seizures; NMDAR: N-methyl-D-aspartate-receptor; NMOSD: neuromyelitis optica spectrum disorder; CNS: central nervous system.

**Table 2:** MOG antibody testing recommendations.

Specificity	MOG-IgG Testing Using a Cell-Based Assay with Full-Length MOG Yields Highly Specific Results.
Preferred specimen type	Testing in serum is optimal; however, CSF testing might be considered in highly suspected seronegative cases.
Timing of testing	Testing is best to be done during attacks and prior to immunotherapy.
Patient selection	Testing should be done only in patients with clinical or imaging features consistent with MOGAD.
Routine Screening	Routine screening of all multiple sclerosis (MS) patients for MOG-IgG is not advisable, as it can heighten the risk of false positives.
False positives	False positives are known to occur, especially at low titers and when the antibody test is requested in low-probability situations.

MS: multiple sclerosis; MOGAD: myelin oligodendrocyte glycoprotein antibody disease; CSF: cerebrospinal fluid.

**Table 3:** Demographic, clinical, and paraclinical characteristics in MOGAD, AQP4 + NMSOD and MS.

	MOGAD	AQP4 + NMOSD	MS
Demographics			
Age at onset	Fourth decade of life	Fourth or fifth decade of life	End of third decade of life
Sex	Slightly more predominant in women (1.5:1)	Female: Male = 9:1	Female: Male = 3:1
Race	No specific differences among different ethnic groups	Afro-Caribbean or Afro-American	White ethnic groups of European descent, although increasing prevalence in all ethnic/racial groups
Clinical Features			
Course	65% monophasic, 35% relapsing (relapses mostly manifest as optic neuritis)	Relapsing	Mostly relapsing, but can be progressive from the beginning
Clinical presentation	Optic neuritis, transverse myelitis, ADEM or other type of encephalitis, FLAMES, brainstem demyelination	Optic neuritis with poor recovery, severe transverse myelitis, area postrema syndrome, brainstem, diencephalic or cerebral syndrome	Spinal cord, brainstem or cerebellar syndrome. Optic neuritis or cognitive dysfunction
Outcome	Mostly favorable outcomes with low risk of future disability. In up to 60–70% of patients with disability, disability comes from the first attack	High risk of disability. Poor prognosis and high relapse rate	High risk of disability accumulation because of relapse associated worsening and progression independent of relapses
MRI			
Optic Nerve	Bilateral optic nerve involvement, anterior optic pathway. More than 50% of the optic nerve is enhancing. Optic disc edema	Bilateral optic nerve involvement, posterior optic pathway including optic chiasm. More than 50% of the optic nerve is enhancing	Unilateral optic nerve involvement. Short lesions
Brain	Heterogenous imaging features. ADEM-like, poorly demarcated, fluffy white matter, deep grey matter lesions, cortical lesions, infratentorial lesions particularly within the cerebellar peduncles and pons. Can be entirely normal	Can be entirely normal. Also, can have deep white matter lesions, or large hemispheric lesions or lesions adjacent to the third or fourth ventricle where AQP4 is mostly expressed	Ovoid lesions perpendicular to the corpus callosum, lesions adjacent to the lateral ventricles, deep white matter lesions or brainstem lesions
Spinal cord	LETM or shorter lesions. Confined to the grey matter (H-shaped). Common involvement of the conus medullaris. Infrequent enhancement	LETM (>3 vertebral segments). Centrally located but not H-shaped. Frequently contrast enhancing	Short lesions peripherally located involving the dorsal or lateral columns. Frequent enhancement
CSF			
White blood cells	Pleocytosis (100–1000 white blood cells/mm—mostly lymphocytes) in 50% of the patients	Pleocytosis (100–1000 cells—mostly lymphocytes) in 50% of the patients	Pleocytosis not so common < 50% of the patients
Biochemistry	Oligoclonal bands < 10%	Oligoclonal bands < 10%	Oligoclonal bands very common > 85–90%
	Elevated protein	Elevated protein	Protein can be elevated or within normal range

LETM: longitudinal extensive transverse myelitis, ADEM: acute disseminated encephalomyelitis; FLAMES: FLAIR-hyperintense Lesions in Anti-MOG-associated Encephalitis with Seizures; NMDAR: N-methyl-D-aspartate-receptor; NMOSD: neuromyelitis optica spectrum disorder; MS: multiple sclerosis; MOGAD: myelin oligodendrocyte glycoprotein antibody disease; CSF: cerebrospinal fluid; AQP4: aquaporin 4.

## Data Availability

Not applicable.

## References

[1] Schluesener H., Sobel R.A., Linington C., Weiner H.L. (1987). A monoclonal antibody against a myelin oligodendrocyte glycoprotein induces relapses and demyelination in central nervous system autoimmune disease. J. Immunol..

[2] Reindl M., Di Pauli F., Rostásy K., Berger T. (2013). The spectrum of MOG autoantibody-associated demyelinating diseases. Nat. Rev. Neurol..

[3] Johns T.G., Bernard C.C. (1999). The structure and function of myelin oligodendrocyte glycoprotein. J. Neurochem..

[4] Hemmer B., Archelos J.J., Hartung H.P. (2002). New concepts in the immunopathogenesis of multiple sclerosis. Nat. Rev. Neurosci..

[5] Cobo-Calvo A., Ruiz A., Maillart E., Audoin B., Zephir H., Bourre B., Ciron J., Collongues N., Brassat D., Cotton F. (2018). Clinical spectrum and prognostic value of CNS MOG autoimmunity in adults: The MOGADOR study. Neurology.

[6] Hacohen Y., Mankad K., Chong W.K., Barkhoff F., Vincent A., Lim M., Wassmer E., Ciccarelli O., Hemingway C. (2017). Diagnostic algorithm for relapsing acquired demyelinating syndromes in children. Neurology.

[7] Flach A.C., Litke T., Strauss J., Haberl M., Gomez C.C., Reindl M., Saiz A., Fehling H.J., Wienands H., Odoardi F. (2016). Autoantibody-boosted T-cell reactivation in the target organ triggers manifestation of autoimmune CNS disease. Proc. Natl. Acad. Sci. USA.

[8] Kinzel S., Lehmann-Horn K., Torke S., Hausler D., Winkler A., Stadelmann C., Payne N., Feldmann L., Saiz A., Reindl M. (2016). Myelin-reactive antibodies initiate T cell-mediated CNS autoimmune disease by opsonization of endogenous antigen. Acta Neuropathol..

[9] Kaneko K., Sato D.K., Nakashima I., Ogawa R., Akaishi T., Takai Y., Nishiyama S., Takahashi T., Misu T., Kuroda H. (2018). CSF cytokine profile in MOG-IgG+ neurological disease is similar to AQP4-IgG+ NMOSD but distinct from MS: A cross-sectional study and potential therapeutic implications. J. Neurol. Neurosurg. Psychiatry.

[10] Mariotto S., Gajofatto A., Batzu L., Delogu R., Sechi R., Leoni S., Pirastru M.I., Bonetti B., Zanoni M., Alberti D. (2019). Relevance of antibodies to myelin oligodendrocyte glycoprotein in CSF of seronegative cases. Neurology.

[11] Spadaro M., Winklmeier S., Beltrán E., Macrini C., Hoftberger R., Schuh E., Thaler F.S., Gerdes L.A., Laurent S., Gerhards  R. (2018). Pathogenicity of human antibodies against myelin oligodendrocyte glycoprotein. Ann. Neurol..

[12] Takai Y., Misu T., Kaneko K., Chihara N., Narikawa K., Tsuchida S., Nishida H., Komori T., Seki M., Komatsu T. (2020). Myelin oligodendrocyte glycoprotein antibody-associated disease: An immunopathological study. Brain.

[13] Mariotto S., Ferrari S., Monaco S., Benedetti M.D., Schanda K., Alberti D., Farinazzo A., Capra R., Mancinelli C., De Rossi N. (2017). Clinical spectrum and IgG subclass analysis of anti-myelin oligodendrocyte glycoprotein antibody-associated syndromes: A multicenter study. J. Neurol..

[14] Ramanathan S., Mohammad S., Tantsis E., Nguyen T.K., Merheb V., Fung V.S.C., White O.B., Broadley S., Lechner-Scott J., Vucic S. (2018). Clinical course, therapeutic responses and outcomes in relapsing MOG antibody-associated demyelination. J. Neurol. Neurosurg. Psychiatry.

[15] Marignier R., Hacohen Y., Cobo-Calvo A., Pröbstel A.K., Aktas O., Alexopoulos H., Amato M.P., Asgari N., Banwell B., Bennett J. (2021). Myelin-oligodendrocyte glycoprotein antibody-associated disease. Lancet Neurol..

[16] Hor J.Y., Fujihara K. (2023). Epidemiology of myelin oligodendrocyte glycoprotein antibody-associated disease: A review of prevalence and incidence worldwide. Front. Neurol..

[17] Sechi E., Cacciaguerra L., Chen J.J., Mariotto S., Fadda G., Dinoto A., Lopez-Chiriboga A.S., Pittock S.J., Flanagan E.P. (2022). Myelin Oligodendrocyte Glycoprotein Antibody-Associated Disease (MOGAD): A Review of Clinical and MRI Features, Diagnosis, and Management. Front. Neurol..

[18] Flanagan E.P., Cabre P., Weinshenker B.G., Sauver J.S., Jacobson D.J., Majed M., Lennon V.A., Lucchinetti C.F., McKeon A., Matiello M. (2016). Epidemiology of aquaporin-4 autoimmunity and neuromyelitis optica spectrum. Ann. Neurol..

[19] De Mol C.L., Wong Y.Y.M., Van Pelt E.D., Wokke B.H.A., Siepman T.A.M., Neuteboom R.F., Hamann D., Hintzen R.Q. (2020). The clinical spectrum and incidence of anti-MOG-associated acquired demyelinating syndromes in children and adults. Mult. Scler..

[20] Sepúlveda M., Aldea M., Escudero D., Llufriu S., Arrambide G., Otero-Romero S., Sastre-Garriga J., Romero-Pinel L., Martínez-Yélamos S., Sola-Valls N. (2017). Epidemiology of NMOSD in Catalonia: Influence of the new 2015 criteria in incidence and prevalence estimates. Mult. Scler..

[21] Hennes E.M., Baumann M., Schanda K., Anlar B., Bajer-Kornek B., Blaschek A., Brantner-Inthaler S., Diepold K., Eisenkölbl A., Gotwald T. (2017). Prognostic relevance of MOG antibodies in children with an acquired demyelinating syndrome. Neurology.

[22] de Mol C.L., Wong Y.Y.M., van Pelt E.D., Ketelslegers I.A., Bakker D.P., Boon M., Braun K.P.J., van Dijk K.G.J., Eikelenboom M.J., Engelen M. (2018). Incidence and outcome of acquired demyelinating syndromes in Dutch children: Update of a nationwide and prospective study. J. Neurol..

[23] Armangue T., Olivé-Cirera G., Martínez-Hernandez E., Sepulveda M., Ruiz-Garcia R., Muñoz-Batista M., Ariño H., González-Álvarez V., Felipe-Rucián A., Jesús Martínez-González M. (2020). Associations of paediatric demyelinating and encephalitic syndromes with myelin oligodendrocyte glycoprotein antibodies: A multicentre observational study. Lancet Neurol..

[24] Boesen M.S., Jensen P.E.H., Born A.P., Magyari M., Nilsson A.C., Hoei-Hansen C., Blinkenberg M., Sellebjerg F. (2019). Incidence of pediatric neuromyelitis optica spectrum disorder and myelin oligodendrocyte glycoprotein antibody-associated disease in Denmark 2008–2018: A nationwide, population-based cohort study. Mult. Scler. Relat. Disord..

[25] Waters P., Fadda G., Woodhall M., O’Mahony J., Brown R.A., Castro D.A., Longoni G., Irani S.R., Sun B., Yeh E. (2020). Serial anti-myelin oligodendrocyte glycoprotein antibody analyses and outcomes in children with demyelinating syndromes. JAMA Neurol..

[26] Cobo-Calvo Á., d’Indy H., Ruiz A., Collongues N., Kremer L., Durand-Dubief F., Rollot F., Casey R., Vukusic S., De Seze J. (2019). Frequency of myelin oligodendrocyte glycoprotein antibody in multiple sclerosis: A multicenter cross-sectional study. Neurol. Neuroimmunol. Neuroinflamm..

[27] Kunchok A., Chen J.J., McKeon A., Mills J.R., Flanagan E.P., Pittock S.J. (2020). Coexistence of myelin oligodendrocyte glycoprotein and aquaporin-4 antibodies in adult and pediatric patients. JAMA Neurol..

[28] Cobo-Calvo A., Ruiz A., Rollot F., Arrambide G., Deschamps R., Maillart E., Papeix C., Audoin B., Lépine A.F., Maurey H. (2021). Clinical features and risk of relapse in children and adults with myelin oligodendrocyte glycoprotein antibody-associated disease. Ann. Neurol..

[29] Baumann M., Hennes E.M., Schanda K., Karenfort M., Kornek B., Seidl R., Diepold K., Lauffer H., Marquardt I., Strautmanis J. (2016). Children with multiphasic disseminated encephalomyelitis and antibodies to the myelin oligodendrocyte glycoprotein (MOG): Extending the spectrum of MOG antibody positive diseases. Mult. Scler..

[30] Satukijchai C., Mariano R., Messina S., Sa M., Woodhall M.R., Robertson N.P., Ming L., Wassmer E., Kneen R., Huda S. (2022). Factors associated with relapse and treatment of myelin oligodendrocyte glycoprotein antibody-associated disease in the United Kingdom. JAMA Netw. Open.

[31] Klein da Costa B., Banwell B.L., Sato D.K. (2021). Treatment of MOG-IgG associated disease in paediatric patients: A systematic review. Mult. Scler. Relat. Disord..

[32] Jurynczyk M., Messina S., Woodhall M.R., Raza N., Everett R., Roca-Fernandez A., Tackley G., Hamid S., Sheard A., Reynolds G. (2017). Clinical presentation and prognosis in MOG-antibody disease: A UK study. Brain.

[33] Yeh E.A., Graves J.S., Benson L.A., Wassmer E., Waldman A. (2016). Pediatric optic neuritis. Neurology.

[34] Chen J.J., Flanagan E.P., Jitprapaikulsan J., López-Chiriboga A.S.S., Fryer J.P., Leavitt J.A., Weinshenker B.G., McKeon A., Tillema J.M., Lennon V.A. (2018). Myelin oligodendrocyte glycoprotein antibody-positive optic neuritis: Clinical characteristics, radiologic clues, and outcome. Am. J. Ophthalmol..

[35] Chen J.J., Bhatti M.T. (2020). Clinical phenotype, radiological features, and treatment of myelin oligodendrocyte glycoprotein-immunoglobulin G (MOG-IgG) optic neuritis. Curr. Opin. Neurol..

[36] Jitprapaikulsan J., Chen J.J., Flanagan E.P., Tobin W.O., Fryer J.P., Weinshenker B.G., McKeon A., Lennon V.A., Leavitt J.A., Tillema J.M. (2018). Aquaporin-4 and Myelin Oligodendrocyte Glycoprotein Autoantibody Status Predict Outcome of Recurrent Optic Neuritis. Ophthalmology.

[37] Oertel F.C., Sotirchos E.S., Zimmermann H.G., Motamedi S., Specovius S., Asseyer E.S., Chien C., Cook L., Vasileiou E., Filippatou A. (2022). Longitudinal Retinal Changes in MOGAD. Ann. Neurol..

[38] Pakeerathan T., Havla J., Schwake C., Salmen A., Bigi S., Abegg M., Brügger D., Ferrazzini T., Runge A.K., Breu M. (2022). Characteristic retinal atrophy pattern allows differentiation between pediatric MOGAD and MS after a single optic neuritis episode. J. Neurol..

[39] Fadda G., Flanagan E.P., Cacciaguerra L., Jitprapaikulsan J., Solla P., Zara P., Sechi E. (2022). Myelitis features and outcomes in CNS demyelinating disorders: Comparison between multiple sclerosis, MOGAD, and AQP4-IgG-positive NMOSD. Front. Neurol..

[40] Mariano R., Messina S., Kumar K., Kuker W., Leite M.I., Palace J. (2019). Comparison of Clinical Outcomes of Transverse Myelitis Among Adults With Myelin Oligodendrocyte Glycoprotein Antibody vs. Aquaporin-4 Antibody Disease. JAMA Netw. Open.

[41] Ciron J., Cobo-Calvo A., Audoin B., Bourre B., Brassat D., Cohen M., Collongues N., Deschamps R., Durand-Dubief F., Laplaud D. (2020). Frequency and characteristics of short versus longitudinally extensive myelitis in adults with MOG antibodies: A retrospective multicentric study. Mult. Scler..

[42] Krupp L.B., Tardieu M., Amato M.P., Banwell B., Chitnis T., Dale R.C., Ghezzi A., Hintzen R., Kornberg A., Pohl D. (2013). International Pediatric Multiple Sclerosis Study Group criteria for pediatric multiple sclerosis and immune-mediated central nervous system demyelinating disorders: Revisions to the 2007 definitions. Mult. Scler..

[43] Wendel E.M., Thonke H.S., Bertolini A., Baumann M., Blaschek A., Merkenschlager A., Karenfort M., Kornek B., Lechner C., Pohl D. (2022). Temporal Dynamics of MOG Antibodies in Children With Acquired Demyelinating Syndrome. Neurol. Neuroimmunol. Neuroinflamm..

[44] Ogawa R., Nakashima I., Takahashi T., Kaneko K., Akaishi T., Takai Y., Sato D.K., Nishiyama S., Misu T., Kuroda H. (2017). MOG antibody-positive, benign, unilateral, cerebral cortical encephalitis with epilepsy. Neurol. Neuroimmunol. Neuroinflamm..

[45] Budhram A., Mirian A., Le C., Hosseini-Moghaddam S.M., Sharma M., Nicolle M.W. (2019). Unilateral cortical FLAIR-hyperintense lesions in anti-MOG-associated Encephalitis with seizures (FLAMES): Characterization of a distinct clinico-radiographic syndrome. J. Neurol..

[46] Stamenova S., Redha I., Schmierer K., Garcia M.E. (2021). FLAIR-hyperintense lesions in anti-MOG-associated encephalitis with seizures (FLAMES) unmasked by withdrawal of immunosuppression for Crohn’s disease?. Mult. Scler. Relat. Disord..

[47] Jain K., Cherian A., Thomas B.J.N. (2021). FLAMES: A novel burning entity in MOG IgG associated disease. Mult. Scler. Relat. Disord..

[48] Hokama H., Sakamoto Y., Hayashi T., Hatake S., Takahashi M., Kodera H., Kutsuna A., Nito C., Nakane S., Nagayama H. (2022). A case report of FLAMES with elevated myelin basic protein followed by myelitis. Intern. Med..

[49] Maniscalco G.T., Allegorico L., Alfieri G., Napolitano M., Ranieri A., Renna R., Servillo G., Pezzella M., Capone E., Altomare L. (2021). Anti-MOG-associated demyelinating disorders: Two sides of the same coin. Neurol. Sci..

[50] Lopez Chiriboga S., Flanagan E.P. (2021). Myelitis and other autoimmune myelopathies. Continuum.

[51] Budhram A., Kunchok A., Flanagan E. (2020). Adding FUEL to the FLAMES: FLAIR-Variable Unilateral Enhancement of the Leptomeninges (FUEL) in MOG-IgG-Associated Disease (862).

[52] Yao T., Zeng Q., Xie Y., Bi F., Zhang L., Xiao B., Zhou J. (2022). Clinical analysis of adult MOG antibody-associated cortical encephalitis. Mult. Scler. Relat. Disord..

[53] Titulaer M.J., Höftberger R., Iizuka T., Leypoldt F., McCracken L., Cellucci T., Benson L.A., Shu H., Irioka T., Hirano M. (2014). Overlapping demyelinating syndromes and anti–N-methyl-D-aspartate receptor encephalitis. Ann. Neurol..

[54] Reindl M., Jarius S., Rostasy K., Berger T. (2017). Myelin oligodendrocyte glycoprotein antibodies: How clinically useful are they?. Curr. Opin. Neurol..

[55] Jarius S., Kleiter I., Ruprecht K., Asgari N., Pitarokoili K., Borisow N., Hümmert M.W., Trebst C., Pache F., Winkelmann A. (2016). MOG-IgG in NMO and related disorders: A multicenter study of 50 patients. Part 3: Brainstem involvement—Frequency, presentation and outcome. J. Neuroinflamm..

[56] Kunchok A., Krecke K.N., Flanagan E.P., Jitprapaikulsan J., Lopez-Chiriboga A.S., Chen J.J., Weinshenker B.G., Pittock S.J. (2020). Does area postrema syndrome occur in myelin oligodendrocyte glycoprotein-IgG-associated disorders (MOGAD)?. Neurology.

[57] Paviolo J.P., Tkachuk V.A. (2020). Isolated area postrema syndrome with anti-MOG antibodies, a rare association. Rev. Neurol..

[58] Chan K.H., Vorobeychik G. (2020). Area postrema syndrome: A neurological presentation of nausea, vomiting and hiccups. BMJ Case Rep..

[59] Akaishi T., Konno M., Nakashima I., Aoki M. (2016). Intractable hiccup in demyelinating disease with anti-myelin oligodendrocyte glycoprotein (MOG) antibody. Intern. Med..

[60] Carnero Contentti E., Rojas J.I., Criniti J., Lopez P.A., Daccach Marques V., Soto de Castillo I., Tkachuk V., Marrodan M., Correale J., Farez M.F. (2022). Towards imaging criteria that best differentiate MS from NMOSD and MOGAD: Large multi-ethnic population and different clinical scenarios. Mult. Scler. Relat. Disord..

[61] Baumann M., Grams A., Djurdjevic T., Wendel E.M., Lechner C., Behring B., Blaschek A., Diepold K., Eisenkölbl A., Fluss J. (2018). MRI of the first event in pediatric acquired demyelinating syndromes with antibodies to myelin oligodendrocyte glycoprotein. J. Neurol..

[62] Juryńczyk M., Tackley G., Kong Y., Geraldes R., Matthews L., Woodhall M., Waters P., Kuker W., Craner M., Weir A. (2017). Brain lesion distribution criteria distinguish MS from AQP4-antibody NMOSD and MOG-antibody disease. J. Neurol. Neurosurg. Psychiatry.

[63] Jurynczyk M., Geraldes R., Probert F., Woodhall M.R., Waters P., Tackley G., DeLuca G., Chandratre S., Leite M.I., Vincent A. (2017). Distinct brain imaging characteristics of autoantibody-mediated CNS conditions and multiple sclerosis. Brain.

[64] Dubey D., Pittock S.J., Krecke K.N., Morris P.P., Sechi E., Zalewski N.L., Weinshenker B.G., Shosha E., Lucchinetti C.F., Fryer J.P. (2019). Clinical, Radiologic, and prognostic features of myelitis associated with myelin oligodendrocyte glycoprotein autoantibody. JAMA Neurol..

[65] Hacohen Y., Rossor T., Mankad K., Chong W., Lux A., Wassmer E., Lim M., Barkhof F., Ciccarelli O., Hemingway C. (2018). ‘Leukodystrophy-like’ phenotype in children with myelin oligodendrocyte glycoprotein antibody-associated disease. Dev. Med. Child. Neurol..

[66] Waters P.J., Komorowski L., Woodhall M., Lederer S., Majed M., Fryer J., Mills J., Flanagan E.P., Irani S.R., Kunchok A.C. (2019). A multicenter comparison of MOG-IgG cell-based assays. Neurology.

[67] Yeh E.A., Nakashima I. (2019). Live-cell based assays are the gold standard for anti-MOG-Ab testing. Neurology.

[68] Sechi E., Buciuc M., Pittock S.J., Chen J.J., Fryer J.P., Jenkins S.M., Budhram A., Weinshenker B.G., Lopez-Chiriboga A.S., Tillema J.M. (2021). Positive Predictive Value of Myelin Oligodendrocyte Glycoprotein Autoantibody Testing. JAMA Neurol..

[69] Held F., Kalluri S.R., Berthele A., Klein A.K., Reindl M., Hemmer B. (2021). Frequency of myelin oligodendrocyte glycoprotein antibodies in a large cohort of neurological patients. Mult. Scler. J. Exp. Transl. Clin..

[70] Jarius S., Ruprecht K., Stellmann J.P., Huss A., Ayzenberg I., Willing A., Trebst C., Pawlitzki M., Abdelhak A., Grüter T. (2018). MOG-IgG in primary and secondary chronic progressive multiple sclerosis: A multicenter study of 200 patients and review of the literature. J. Neuroinflamm..

[71] Jarius S., Paul F., Aktas O., Asgari N., Dale R.C., de Seze J., Franciotta D., Fujihara K., Jacob A., Kim H.J. (2018). MOG encephalomyelitis: International recommendations on diagnosis and antibody testing. J. Neuroinflamm..

[72] Banwell B., Bennett J.L., Marignier R., Kim H.J., Brilot F., Flanagan E.P., Ramanathan S., Waters P., Tenembaum S., Graves J.S. (2023). Diagnosis of myelin oligodendrocyte glycoprotein antibody-associated disease: International MOGAD Panel proposed criteria. Lancet Neurol..

[73] Hegen H., Reindl M. (2020). Recent developments in MOG-IgG associated neurological disorders. Ther. Adv. Neurol. Disord..

[74] Chen B., Gomez-Figueroa E., Redenbaugh V., Francis A., Satukijchai C., Wu Y., Messina S., Sa M., Woodhall M., Paul F. (2023). Do Early Relapses Predict the Risk of Long-Term Relapsing Disease in an Adult and Paediatric Cohort with MOGAD?. Ann. Neurol..

[75] Leite M.I., Panahloo Z., Harrison N., Palace J. (2023). A systematic literature review to examine the considerations around pregnancy in women of child-bearing age with myelin oligodendrocyte glycoprotein antibody-associated disease (MOGAD) or aquaporin 4 neuromyelitis optica spectrum disorder (AQP4+ NMOSD). Mult. Scler. Relat. Disord..

[76] Jarius S., Ruprecht K., Kleiter I., Borisow N., Asgari N., Pitarokoili K., Pache F., Stich O., Beume L.A., Hümmert M.W. (2016). MOG-IgG in NMO and related disorders: A multicenter study of 50 patients. Part 2: Epidemiology, clinical presentation, radiological and laboratory features, treatment responses, and long-term outcome. J. Neuroinflamm..

[77] Hacohen Y., Wong Y.Y., Lechner C., Jurynczyk M., Wright S., Konuskan B., Kalser J., Poulat A.L., Maurey H., Ganelin-Cohen E. (2018). Disease course and treatment responses in children with relapsing myelin oligodendrocyte glycoprotein antibody-associated disease. JAMA Neurol..

[78] Zhou J., Lu X., Zhang Y., Ji T., Jin Y., Xu M., Bao X., Zhang Y., Xiong H., Chang X. (2019). Follow-up study on Chinese children with relapsing MOG-IgG-associated central nervous system demyelination. Mult. Scler. Relat. Disord..

[79] Chen J.J., Flanagan E.P., Bhatti M.T., Jitprapaikulsan J., Dubey D., Lopez Chiriboga A.S.S., Fryer J.P., Weinshenker B.G., McKeon A., Tillema J.M. (2020). Steroid-sparing maintenance immunotherapy for MOG-IgG associated disorder. Neurology.

[80] Cobo-Calvo A., Sepúlveda M., Rollot F., Armangué T., Ruiz A., Maillart E., Papeix C., Audoin B., Zephir H., Biotti D. (2019). Evaluation of treatment response in adults with relapsing MOG-Ab-associated disease. J. Neuroinflamm..

[81] Cobo-Calvo A., Sepúlveda M., Rollot F., Armangué T., Ruiz A., Maillart E., Papeix C., Audoin B., Zephir H., Biotti D. (2021). A comparison of the effects of rituximab versus other immunotherapies for MOG-IgG-associated central nervous system demyelination: A meta-analysis. Mult. Scler. Relat. Disord..

[82] Elsbernd P.M., Hoffman W.R., Carter J.L., Wingerchuk D.M. (2021). Interleukin-6 inhibition with tocilizumab for relapsing MOG-IgG associated disorder (MOGAD): A case-series and review. Mult. Scler. Relat. Disord..

[83] Bril V., Drużdż A., Grosskreutz J., Habib A.A., Mantegazza R., Sacconi S., Utsugisawa K., Vissing J., Vu T., Boehnlein M. (2023). Safety and efficacy of rozanolixizumab in patients with generalised myasthenia gravis (MycarinG): A randomised, double-blind, placebo-controlled, adaptive phase 3 study. Lancet Neurol..

